# Generation and characterization of transgene-free human induced pluripotent stem cells and conversion to putative clinical-grade status

**DOI:** 10.1186/scrt246

**Published:** 2013-07-26

**Authors:** Jason P Awe, Patrick C Lee, Cyril Ramathal, Agustin Vega-Crespo, Jens Durruthy-Durruthy, Aaron Cooper, Saravanan Karumbayaram, William E Lowry, Amander T Clark, Jerome A Zack, Vittorio Sebastiano, Donald B Kohn, April D Pyle, Martin G Martin, Gerald S Lipshutz, Patricia E Phelps, Renee A Reijo Pera, James A Byrne

**Affiliations:** 1 Department of Molecular and Medical Pharmacology, 23–120 Center for Health Sciences, University of California, Los Angeles (UCLA), 650 Charles E. Young Drive South, Los Angeles, CA 90095, USA; 2 Department of Obstetrics and Gynecology, Institute for Stem Cell Biology & Regenerative Medicine, Stanford University School of Medicine, Stanford University, 265 Campus Drive, Stanford, Stanford, CA 94305, USA; 3 Department of Microbiology, Immunology and Molecular Genetics, 609 Charles E. Young Drive East, UCLA, Los Angeles, CA 90095, USA; 4 Eli and Edythe Broad Center of Regenerative Medicine and Stem Cell Research, 615 Charles E Young Drive South, UCLA, Los Angeles, CA 90095, USA; 5 Department of Pediatrics, Division of Gastroenterology and Nutrition, 10833 Le Conte Ave, UCLA, Los Angeles, CA 90095, USA; 6 Department of Surgery, 200 UCLA Medical Plaza, UCLA, Los Angeles, CA 90095, USA; 7 Department of Integrative Biology and Physiology, 612 Charles E. Young Drive East, UCLA, Los Angeles, CA 90095, USA

## Abstract

**Introduction:**

The reprogramming of a patient’s somatic cells back into induced pluripotent stem cells (iPSCs) holds significant promise for future autologous cellular therapeutics. The continued presence of potentially oncogenic transgenic elements following reprogramming, however, represents a safety concern that should be addressed prior to clinical applications. The polycistronic stem cell cassette (STEMCCA), an excisable lentiviral reprogramming vector, provides, in our hands, the most consistent reprogramming approach that addresses this safety concern. Nevertheless, most viral integrations occur in genes, and exactly how the integration, epigenetic reprogramming, and excision of the STEMCCA reprogramming vector influences those genes and whether these cells still have clinical potential are not yet known.

**Methods:**

In this study, we used both microarray and sensitive real-time PCR to investigate gene expression changes following both intron-based reprogramming and excision of the STEMCCA cassette during the generation of human iPSCs from adult human dermal fibroblasts. Integration site analysis was conducted using nonrestrictive linear amplification PCR. Transgene-free iPSCs were fully characterized via immunocytochemistry, karyotyping and teratoma formation, and current protocols were implemented for guided differentiation. We also utilized current good manufacturing practice guidelines and manufacturing facilities for conversion of our iPSCs into putative clinical grade conditions.

**Results:**

We found that a STEMCCA-derived iPSC line that contains a single integration, found to be located in an intronic location in an actively transcribed gene, *PRPF39*, displays significantly increased expression when compared with post-excised stem cells. STEMCCA excision via Cre recombinase returned basal expression levels of *PRPF39*. These cells were also shown to have proper splicing patterns and *PRPF39* gene sequences. We also fully characterized the post-excision iPSCs, differentiated them into multiple clinically relevant cell types (including oligodendrocytes, hepatocytes, and cardiomyocytes), and converted them to putative clinical-grade conditions using the same approach previously approved by the US Food and Drug Administration for the conversion of human embryonic stem cells from research-grade to clinical-grade status.

**Conclusion:**

For the first time, these studies provide a proof-of-principle for the generation of fully characterized transgene-free human iPSCs and, in light of the limited availability of current good manufacturing practice cellular manufacturing facilities, highlight an attractive potential mechanism for converting research-grade cell lines into putatively clinical-grade biologics for personalized cellular therapeutics.

## Introduction

Previous research demonstrated that human somatic cells can be directly reprogrammed back into an induced pluripotent stem cell (iPSC) state through exogenous expression of a small number of transgenic factors [1]. The ability of these cells to differentiate into any human cell type highlights their promise for future autologous cellular therapies [2,3]. Nevertheless, the continued presence of potentially oncogenic transgenic elements following reprogramming represents a safety concern that must be addressed prior to clinical applications [4-7]. Various integration-free approaches have been investigated to address this safety concern. Of the various techniques tested to date – that is, episomal plasmids [8], minicircles [9], nonintegrating miRNAs [10,11], cell-permeable proteins [12], sendai viruses [13], synthetic mRNAs [14] and the removable polycistronic stem cell cassette (STEMCCA) – and despite each having published reprogramming success (Table 1), only the STEMCCA-based reprogramming approach, in our hands, has consistently and successfully reprogrammed dermal fibroblasts from multiple different adult donors into iPSCs.

**Table 1:** Human induced pluripotent stem cell reprogramming efficiencies from human dermal fibroblasts

	**Lentivirus**	**Polycistronic STEMCCA lentivirus**	**Retrovirus**	**Episomal / minicircle**	**miRNAs**	**Cell-permeable proteins**	**Sendai virus**	**mRNA**
Efficiency (%)	0.022	0.01 to 1.5	<0.01 to 0.02	0.003 to 0.006	0.002	0.001	0.01 to 1	>1
Integrating	Yes	Yes	Yes	No	No	No	No	No
Reference(s)	[1]	[15,16]	[17,18]	[8,9]	[11]	[12]	[13]	[14]

Advantages of the STEMCCA reprogramming approach include the following: lentiviruses can transduce both dividing and nondividing cells; the STEMCCA polycistronic cassette was engineered for efficient production of multiple protein products from a single lentivirus and allows a characteristic stoichiometry of protein expression that reproducibly promotes consistent reprogramming success [15,19]; the STEMCCA approach involves only a single transduction event, making it less labor intensive than more involved reprogramming methods such as synthetic mRNAs; the STEMCCA cassette is excisable, eliminating residual transgene expression that reportedly compromises differentiation potential [20]; and iPSCs can be generated to contain only one integration event and accurately mapped in the genome [16,20,21]. To date, a variety of cell types have been reprogrammed through polycistronic lentivirus-mediated reprogramming, including human keratinocytes, bone marrow cells, skin fibroblasts [22], and T cells from peripheral blood [23] and also from patients with diseases such as Huntington’s disease [24], heart failure [25], immunodeficiency disorders [26], lung disease [16], and neurodevelopmental disorders [27]. Nevertheless, the majority (approximately 70%) of lentiviral integrations occur in actively transcribed genes [28,29]. Because current safe-harbor criteria discard iPSC lines that result from a viral integration occurring in a gene [30], this greatly reduces the feasibility of STEMCCA-iPSC-based therapeutics. We and others have previously relied solely on microarray transcriptional analysis to assess the expression of genes following insertion of STEMCCA into the introns of genes [30,31].

In this study, we use both microarray and sensitive real-time RT-PCR to investigate gene expression changes following both intron-based integration and excision of the STEMCCA cassette during the generation of human induced pluripotent stem cells (hiPSCs). We also fully characterized the post-excised iPSCs, differentiated them into four therapeutically useful cell types, and converted them into putative clinical-grade conditions.

## Materials and methods

### Ethics statement

Written approvals for human skin biopsy procedures and human fibroblast derivation, culture, and experimental use were obtained from the Stanford University Institutional Review Board (Stanford IRB protocol #10368) and the Stanford University Stem Cell Research Oversight Committee (Stanford SCRO protocol #40), and written informed consent was obtained from each individual participant. Cells used in this study were initially derived at Stanford University and transferred to UCLA through a material transfer agreement (UCLA MTA #2011-00000147). Written approvals for the experiments performed in this study were obtained from the UCLA Institute Biosafety Committee (UCLA IBC protocol #123.10.0-f), the Animal Research Committee (UCLA ARC protocol #2006-119-21) and the Stem Cell Research Oversight Committee (UCLA SCRO protocol #2010-010-02).

### *In vitro* culture of primary human skin cells

The human skin-derived (HUF1) primary cell line used in this study was obtained from a 4-mm adult skin punch biopsy and was cultured as described [32]. Two other fibroblast lines were also used in this study: an infant fibroblast line (MGM2) and a fibroblast line from Fibrocell Science, Inc. (Exton, PA, USA) (azficel-T (LAVIV) part #DR01/RMS-5519v00). All human biopsy-derived cells and fibroblast lines were cultured in complete DMEM/F-12 media consisting of DMEM nutrient mixture/F-12 supplemented with 10% fetal bovine serum (FBS), 1× minimum essential medium nonessential amino acid, 1× Glutamax, and 100 IU/ml penicillin–streptomycin (all from Invitrogen/Gibco, Grand Island, NY, USA) and maintained at 37°C in a 5% CO_2_ incubator. Culture media were changed every 2 days. Cells were allowed to expand to 80 to 90% confluency before passaging with 0.05% trypsin–ethylenediamine tetraacetic acid (Invitrogen) and replating at a 1:3 ratio. A large bank of early-passage HUF1 cells was cryopreserved in culture media supplemented with 10% dimethyl sulfoxide (Sigma-Aldrich, St Louis, MO, USA). All research adhered to National Academy of Sciences guidelines.

### *In vitro* culture of stem cell lines

Human-1, human-2, and human-9 embryonic stem cell (ESC) lines were provided by the UCLA Broad Stem Cell Research Center-Stem Cell Core. Multiple integration iPSCs were derived as previously published [31]. The mRNA hiPSCs were derived using Stemgent’s mRNA reprogramming factor set (Stemgent, San Diego, CA, USA). The adult pre-excision line (termed C-8, or pre-excised iPSC) and the adult post-excision line (termed 2.3, or post-excised iPSC), derived as explained below, were all initially maintained on 0.2% gelatin-coated six-well plates covered with 35,000 cells/cm^2^ irradiated mouse embryonic fibroblasts (MEFs) (GlobalStem, Rockville, MD, USA) with standard ESC media consisting of DMEM/F-12 supplemented with 20% Knockout Serum Replacement, 1× Glutamax, 1× nonessential amino acid, 100 IU/ml penicillin–streptomycin (all from Invitrogen), 1× β-mercaptoethanol (Millipore, Billerica, MA, USA), and 10 ng/ml recombinant human basic fibroblast growth factor (Globalstem). All cells were transitioned into a feeder-free system and subsequently maintained on reduced growth factor Matrigel (BD Biosciences, San Jose, CA, USA) in mTeSR1 medium (Stem Cell Technologies, Vancouver, BC, Canada) supplemented with 10 ng/ml basic fibroblast growth factor (Globalstem) and 1× Primocin (InvivoGen, San Diego, CA, USA). Media were changed daily. Cells were passaged every 4 to 5 days, depending on colony density and size. Differentiation was removed daily from colonies using pulled glass pipettes. To passage the pluripotent stem cells, an 18-gauge needle was used to cross-hatch colonies in a grid format, with subsequent gentle agitation to remove the pieces with a P200 pipette. Usually, 4 to 8 colonies were passaged onto freshly coated Matrigel plates.

### Lentivirus production and infection

For pre-excised and post-excised iPSC lines, lentiviral human STEMCCA vector was synthesized and packaged as published [15] and was concentrated to 100×. The day before infection, 100,000 cells/well were plated in a six-well plate grown in standard DMEM/F-12 media without antibiotics. On the day of transduction, 100× lentiviral supernatant was thawed, and 2 ml MEF conditioned media from each well of fibroblasts to be infected was taken out and mixed with 2× and 4× viral supernatant concentrations, respectively, with 8 μg/ml polybrene (Millipore). This virus-containing mixture was quickly added to the cells to avoid drying, shaken gently, and placed at 37°C in a 5% CO_2_ incubator overnight. From day 2 through day 6, media were changed every day with DMEM/F-12 medium with antibiotics. Irradiated xCF1 fibroblasts harvested from day 8 mouse embryos were plated on day 6, and 50,000 and 100,000 cells from one well in a six-well plate were plated on day 7 onto an MEF-plated 10-cm plate and left to sit at 37°C in a 5% CO_2_ incubator overnight. The next day, MEF media were replaced with human ESC medium for the duration of the reprogramming and changed daily. Colonies were picked on the parental plate when colonies reached the size of 60 to 70% of 5× field view or became three-dimensional/differentiated into cell aggregates. Each parental colony was cut into two or three pieces and seeded onto a 24-well plate preseeded with xCF1 mouse feeders, one clone per well. Colonies were grown and further subcloned out according to optimal growth and colony morphology (flattened, very little differentiation, and high nucleus-to-cytoplasm ratio) and when colonies reached 60 to 70% of 5× field. Subcloning into a 12-well plate required 8 to 10 pieces from each clone per well from a 24-well plate be placed into an xCF1 MEF precoated 12-well plate. The pieces were then eventually subcloned out to a six-well plate for further characterization.

### Vector integration site analysis by nonrestrictive linear amplification PCR

DNA was isolated from iPSCs using the PureLink Genomic DNA Mini Kit (Invitrogen). Approximately 100 ng genomic DNA was used to perform nonrestrictive linear amplification (nrLAM) PCR [33]. Briefly, 100 cycles of linear amplification were performed with primer HIV3linear (Biotin-agtagtgtgtgcccgtctgt). Linear reactions were purified using 1.5 volumes of AMPure XP beads (Beckman Genomics, Indianapolis, IN, USA) and captured onto 100 μg of M-280 Streptavidin Dynabeads (Invitrogen Dynal), prepared in accordance with the instructions of the manufacturer. Captured ssDNA was ligated to read 2 linker (Phos-agatcggaagagcacacgtctgaactccagtcac-3C Spacer) using CircLigase II (Epicentre, Madison, WI, USA) in a 10 μl reaction at 65° for 2 hours. PCR was performed on these beads using primer HIV3right (aatgatacggcgaccaccgagatctacactgatccctcagacccttttagtc) and an appropriate indexed reverse primer (caagcagaagacggcatacgagat-index-gtgactggagttcagacgtgt). PCR products were mixed and quantified by probe-based quantitative PCR, and appropriate amounts were used to load Illumina v3 flow cells (Illumina, San Diego, CA, USA). Paired-end 50-base-pair sequencing was performed on an Illumina HiSeq 2000 instrument using a custom read 1 primer (ccctcagacccttttagtcagtgtggaaaatctctagca). Reads were aligned to the hg19 build of the human genome with Bowtie [34], and alignments were condensed and annotated using custom Perl and Python scripts to locate vector integrations.

### Infection of induced pluripotent stem cells with adeno-Cre

Excision of STEMCCA was performed by transient transduction of a defective adenoviral vector expressing Cre-recombinase-puromycin (Adeno-Cre-puroR), which was generated by Vector BioLabs (Philadelphia, PA, USA) to express Cre recombinase and puromycin resistance, into the parental pre-excised iPSC line. We used 45 and 5 μl concentrated Adeno-Cre-puroR virus with 8 μg/ml polybrene (Millipore) in standard ESC media for 24 hours. After 24 hours (on day 1), the mixed viral supernatant was removed, and the cells were washed twice with ESC media and then cultured in fresh ESC media containing 2 μg/ml puromycin (Invitrogen) for a period of 5 days. Individual colonies still growing after 5 days were subcloned into 12-well plates and expanded as described above.

### Genomic and RT-PCR analysis

Genomic DNA was isolated from pluripotent stem cells (PSCs) grown in feeder-free conditions with the PureLink Genomic DNA Mini Kit (Invitrogen) in accordance with the instructions of the manufacturer. PCR was performed using the KAPA HiFi Hotstart ReadyMix PCR kit (KAPA, Woburn, MA, USA) with a five-step PCR protocol as follows: initial denaturation at 95°C for 3 minutes; 35 cycles of each of the following: denaturation at 98°C for 20 seconds, primer annealing at 62°C for 15 seconds, and extension at 72°C for 15 seconds; followed by a single cycle final extension at 72°C for 3 minutes. Ten nanograms of template DNA were used. Primers specific for exogenous integrations of the STEMCCA lentivirus are listed as follows: gDNA-hendo-MycS-forward, 5′-acgagcacaagctcacctct-3′; gDNA-hWPRE-reverse, 5′-tcagcaaacacagtgcacacc-3′. gDNA PCR was normalized to beta-actin: gDNA-hACTB-forward, 5′-ggagaatggcccagtcctc-3′; and gDNA-hACTB-reverse, 5′-ggtctcaagtcagtgtacagg-3′ [20]. Total RNA was isolated using PSCs grown only on feeder-free conditions to prevent MEF mRNA contamination issues with Roche’s High Pure RNA Isolation Kit in accordance with the instructions of the manufacturer (Roche, Indianapolis, IN, USA). Then 700 ng PSCs and 300 ng all fibroblast lines’ RNA were reverse-transcribed using the Transcriptor First Strand cDNA Synthesis Kit, using anchored-oligo(dT)_18_ and random hexamer primers (Roche). PCR was performed using the KAPA HiFi Hotstart ReadyMix PCR kit (KAPA) with a five-step PCR protocol: initial denaturation at 95°C for 5 minutes; 28 cycles of each of the following: denaturation at 98°C for 20 seconds, primer annealing at 64°C for 15 seconds, and extension at 72°C for 15 seconds; followed by a single cycle final extension at 72°C for 5 minutes. In total, 75 ng RNA was used per reaction, and 12 μl with 3 μl loading dye was loaded into a 3% agarose gel in accordance with the recommendations of the manufacturer. Primers specific to exon 4/5 splice junction analysis were: RT-hexon4/5-forward, 5′-tgagcatgctgttctagctgcagga-3′; and RT-hexon4/5-reverse, 5′-accaggaggaccatcatcaccac-3′. RT-PCR gene expression was normalized to beta-actin: RT-hACTB-forward, 5′-ggagaatggcccagtcctc-3′; and RT-hACTB-reverse, 5′-ggtctcaagtcagtgtacagg-3′.

### Global transcriptional meta-analysis

Pre-excised and post-excised iPSCs were grown in standard feeder-free culture conditions as stated above and harvested for total mRNA using a High Pure RNA Isolation Kit in accordance with the instructions of the manufacturer (Roche). Microarray analysis was carried out as published [35]. Affymetrix data adhered to the standards proposed by the Functional Genomics Data Society and were deposited in a MIAME-compliant format into the Gene Expression Omnibus [36] [GEO:GSE48830]. Each CEL file was uploaded to GeneSifter (VisX Labs, Seattle, WA, USA) using the Advanced Upload Method and normalized using the Affymetrix Microarray Analysis Suite (MAS) 5.0 (Santa Clara, CA, USA) algorithm. GeneSifter pairwise analysis between samples was performed using all mean normalization and *t*-test statistical analysis (*P* <0.05). For each pairwise analysis, two replicates from each cell line were compared. Probe sets were considered significantly different when *P* <0.05 and fold change ≥2.

### Quantitative reverse transcription-polymerase chain reaction

Total RNA was isolated using PSCs grown only on feeder-free conditions to prevent MEF mRNA contamination issues as stated above. Primers and probes were designed and ordered from Roche’s Universal ProbeLibrary. Quantitative PCR relative expression experiments used a LightCycler 480 Real-Time PCR System (Roche), and data were further analyzed with LightCycler 480 Software release 1.5.0. Primers for the genes are listed as follows – primers specific for pre-loxP site analysis: QRT-h*PRPF39*-forward, 5′-caggattttacaggctgggta-3′ and QRT-h*PRPF39*-reverse, 5′-tcctggcagccatcaagt-3′, probe #2; QRT-hPOU5F1-forward, 5′-gaagttaggtgggcagcttg-3′ and QRT-hPOU5F1-reverse, 5′-tgtggccccaaggaatagt-3′, probe #13; QRT-hSOX2-forward, 5′-gggggaatggaccttgtatag-3′ and QRT-hSOX2-reverse, 5′-gcaaagctcctaccgtacca-3′, probe #65; QRT-hNANOG-forward, 5′-cagtctggacactggctgaa-3′ and QRT-hNANOG-reverse, 5′-cacgtggtttccaaacaaga-3′, probe #55; and gene expression was normalized using HPRT1 and GAPDH primers: QRT-hHPRT1-forward, 5′-tgaccttgatttattttgcatacc-3′ and QRT-hHPRT1-reverse, 5′-cgagcaagacgttcagtcct-3′, probe #73; and QRT-GAPDH-forward, 5′-gctctctgctcctcctgttc-3′ and QRT-GAPDH-reverse, 5′-acgaccaaatccgttgactc-3′, probe #60. Five nanograms per sample were used in a 20 μl reaction that consisted of 10 μM UPL probe, 2× LightCycler 480 Probes Master, and 20 μM forward and reverse primers. Triplicate experimental samples were paired using the all-to-mean pairing rule with two housekeeping genes run in duplicate for advanced relative quantification.

### Sequencing

Total RNA was extracted as stated above and amplified with the hexon 4/5 primers and purified with a PCR purification kit (Qiagen, Valencia, CA, USA). Samples were sent for full-service sequencing at UCLA’s Genotyping and Sequencing Core (Los Angeles, CA, USA) using Invitrogen/Applied Biosystems 3730 Capillary DNA Analyzers, and sequence results were analyzed on ApE by (M. Wayne Davis; [37]).

### Immunocytochemistry

Cultured cells were fixed in 4% paraformaldehyde/1× PBS for 15 minutes, washed twice with 1× PBS supplemented with 100 mM glycine for 5 minutes, and then incubated, when needed, with permeabilization buffer consisting of 0.1% Triton X-100 (Sigma-Aldrich) in 1× PBS for 30 minutes at room temperature. Blocking was performed with 4% goat serum in Blocker Casein in PBS (Thermo Scientific, Rockford, IL, USA) for 60 minutes at room temperature. The cells were then incubated for 2.5 hours with primary antibody at room temperature. Cells were washed with PBS after primary antibody staining and following each subsequent step. Following primary antibody incubation, the coverslips/wells were incubated with Alexa Fluor secondary antibodies (Invitrogen) at room temperature for 1 hour and mounted in Prolong Gold with 4′,6-diamidino-2-phenylindole (Invitrogen). Cultures were visualized with an AxioCam MR Monocolor Camera and AxioVision Digital Image Processing Software (Axio Observer Inverted Microscope; Carl Zeiss, Jena, Germany).

The primary antibodies used for PSC characterization are mouse anti-Oct-3/4 (C-10) (1:200; Santa Cruz Biotechnology, Santa Cruz, CA, USA), rat anti-SSEA-3 (1:200; Millipore), mouse anti-SSEA-4 (1:200; Millipore), mouse anti-TRA-1-60 (1:200; Millipore), mouse anti-TRA-1-81 (1:200; Millipore), and rabbit anti-NANOG (1:100; Abcam, Cambridge, MA, USA) [32]. For oligodendrocyte progenitor and oligodendrocyte cells, the following primary antibodies were used: mouse anti-NG2 (1:25; eBioscience, San Diego, CA, USA), rabbit anti-PDGFRα (1:20; Abcam), rabbit anti-SOX10 (1:20; Abcam), mouse anti-OLIG1 (1:200; Millipore), mouse anti-A2B5 (1:50; Millipore), mouse anti-O4 (1:40; R&D Systems, Minneapolis, MN, USA), mouse anti-O1 (1:40; R&D Systems), and rat anti-Myelin Basic Protein (1:40; Abcam). To analyze oligodendrocyte and neuronal co-culture, and to ensure oligodendrocyte human origin, rabbit anti-TUJ-1 (1:2500; Covance, Inc., Emeryville, CA, USA) and mouse anti-human mitochondria (1:40; Millipore) antibodies were used, respectively. For hepatocyte cells, the following primary antibodies were used: mouse anti-CK18 (1:50; Dako, Carpinteria, CA, USA), mouse anti-serum albumin (1:50; R&D Systems), and mouse anti-alpha-fetoprotein (1:100; Invitrogen). For cardiomyocytes, the following primary antibodies were used: mouse anti-Troponin I (1:50; Millipore) and mouse anti-alpha-actinin (Sarcomeric) (1:100; Sigma-Aldrich). For fibroblast differentiation, the following primary antibody was used: mouse anti-COL3A1 (1:40; Santa Cruz Biotechnology).

### Induced pluripotent stem cell-directed differentiation

For oligodendrocyte progenitor and mature oligodendrocyte differentiation, embryoid bodies (EBs) were made on day 1 by 1 mg/ml collagenase treatment for 10 minutes, followed by gentle scraping with a 5-ml serological pipette. Detached colonies were collected and transferred to low-adhesion plates (Sigma-Aldrich) in a 50:50 combination of mTeSR1 and Glial Restrictive Media and differentiated as published [38]. For co-culture experiments, rat dorsal root ganglion (DRG) neurons were dissected and cultured as previously described, except for the substitution of rat DRG neurons [39]. DRG neurons were cultured on Matrigel (BD Biosciences) for a period of 7 days before post-excised derived oligodendrocyte progenitor cells were plated on top of the DRG neurons at a density of 15,000 cells/well in a 24-well plate. All cells were cultured in Glial Restrictive Media. Co-cultured cells were cultured for a period of 7 days before fixation and immunostaining.

For EB-directed beating cardiomyocyte differentiation, post-excised iPSCs were incubated with 1 mg/ml collagenase for 10 minutes and then quenched with standard differentiation media consisting of standard DMEM as listed above but with 20% FBS and also with inclusion of 50 μg/ml ascorbic acid (Sigma-Aldrich), followed by making strips of iPSCs with a 5-ml serological pipette and subsequent placement into low-adhesion plates (Sigma-Aldrich). Media were changed every day with fresh media until day 5, when EBs were plated onto 0.2% gelatin-coated plates. The FBS concentration was reduced to 5% on day 10, and media were changed every 4 to 5 days with fresh ascorbic acid [40].

For non-EB-directed cardiomyocyte differentiation, post-excised iPSCs cultured on Matrigel were changed to DMEM/F-12 (Invitrogen) supplemented with 1× N2, 2 mM l-glutamine, 1 mM nonessential amino acid, 1× B27 supplement (all from Invitrogen), 0.5 mg/ml bovine serum albumin (Fraction V; Sigma-Aldrich), and 0.11 mM 2-mercaptoethanol (Millipore) (N2/B27-CDM) supplemented with 50 ng/ml recombinant human BMP-4 and 50 ng/ml recombinant human activin A (both from PeproTech, Rocky Hill, NJ, USA) for 3 or 4 days and cultured in N2/B27-CDM without additional factors for an additional 8 to 10 days. The medium was changed daily [41].

For hepatocyte differentiation, post-excised iPSCs were grown on Matrigel as stated above until reaching a 60 to 70% confluence upon which endoderm induction was initiated by replacing the post-excised iPSCs for 24 hours with RPMI 1640 medium (Invitrogen/Gibco, Rockville, MD, USA), supplemented with 0.5 mg/ml albumin fraction V (Sigma-Aldrich), and 100 ng/ml Activin A (PeproTech). On the following 2 days, 0.1 and 1% insulin–transferrin–selenium (Invitrogen/Gibco) were added to the medium, respectively. Post-excised iPSCs were then cultured in hepatocyte culture medium (Lonza, Walkersville, MD, USA) containing 30 ng/ml fibroblast growth factor-4 and 20 ng/ml BMP2 (PeproTech) for 4 days. The now-differentiated cells were then incubated in hepatocyte culture medium containing 20 ng/ml hematopoietic growth factor and 20 ng/ml keratinocyte growth factor (PeproTech) for 6 days, in hepatocyte culture medium containing 10 ng/ml oncostatin-M (R&D Systems) plus 0.1 μM dexamethasone (Sigma-Aldrich) for 5 days, and in DMEM containing N2, B27, 1× Glutamax, 1× nonessential amino acid, and 1× β-mercaptoethanol (all from Invitrogen/Gibco) for 3 more days. Media were changed daily during differentiation [42].

For fibroblast differentiation, EBs were cultured in adherent conditions on 0.2% gelatin using standard fibroblast media with 10% FBS and were passaged until typical fibroblast morphology was seen [43].

### Karyotype analysis

Post-excised iPSCs were passaged onto a 25-cm^2^ flask to 60 to 70% confluency and sent out for G-band karyotyping analysis (Cell Line Genetics, Madison, WI, USA).

### Teratoma formation

Teratomas for the pre-excised and post-excised iPSC lines were generated by injecting 8 × 10^6^ cells resuspended in Hanks’ balanced salt solution (Invitrogen) into the two testes in a severe combined immunodeficient adult male beige mouse. All tumors were dissected 6 to 8 weeks after injection and fixed in 4% formaldehyde, and sections were paraffin-embedded and then stained with H & E for further analysis at the UCLA Translational Pathology Laboratory. All animal experiments were performed in accordance with the UCLA Animal Research Committee and the UCLA Division of Laboratory Animal Medicine.

### Good manufacturing practice conversion and analysis

Post-excised iPSCs were slowly transitioned from mTeSR1 media conditions to a 1:1 ratio of mTeSR1 and NutriStem (Stemgent) and finally to a 1:1 ratio of TeSR2/NutriStem (STEMCELL Technologies, Vancouver, BC, Canada) supplemented with 1× Primocin (InvivoGen) and 1× basic fibroblast growth factor (GlobalStem), which are both defined xeno-free media (containing no animal proteins). This conversion used 0:100, 20:80, 50:50, 80:20, and 100:0 mTeSR1/NutriStem:TeSR2/NutriStem ratios, with each condition lasting for 3 days. Regular passaging was maintained every 4 or 5 days based on cell morphology and density. Once cells were converted to the 1:1 TeSR2/NutriStem, the cells were mechanically passaged with an 18-gauge needle in the presence of 1× ROCK inhibitor (Stemgent), preconditioned in the media for 1 hour, and then transferred to a xeno-free substrate (Synthemax; Sigma-Aldrich). Cells were initially fibroblastic in nature, and continual differentiation of the iPSCs had to be taken out with a hand-pulled glass pipette. Specific selection of proper iPSC colonies over a period of 2 or 3 weeks generated morphologically homogeneous and standard-looking iPSCs. Cells that were converted to xeno-free conditions were then transferred to the UCLA good manufacturing practice (GMP)-compatible facility and underwent extended cultivation (for over 3 months) under xeno-free conditions. The cells were then subjected to standardized quality-control testing to ensure viability, sterility, and appropriate cellular composition, which included immunocytochemical analysis of stem cell markers, confirmation that the cells were free from nonhuman contaminants, including bacteria, fungi, mycoplasma or sialic acid (Neu5Gc) contamination, and confirmation they possessed a normal karyotype, and were cryobanked for potential future clinical applications as previously described [31]. To further show the broad applicability of our slow transition methodology across media and synthetic matrices, we also converted the post-excised cells to a fully defined, synthetic matrix called CELLstart (Invitrogen) and cultured in NutriStem media alone.

### Flow cytometry-based detection of sialic acid contamination

Flow cytometry was performed on the BD LSRII flow cytometer and all data were analyzed with BD FACSDiva Version 6.1.3 Software (BD Biosciences). The cell surface expression of nonhuman sialic acid Neu5Gc (*N*-glycolylneuraminic acid) was detected utilizing the chicken anti-Neu5Gc IgG (1:200) (Sialix anti-Neu5Gc Basic Pack Kit; Sialix San Diego, CA, USA) and labeled with FITC-conjugated donkey anti-chicken IgG (H + L) (1:200; Jackson ImmunoResearch, West Grove, PA, USA). 4’,6-Diamidino-2-phenylindole (Invitrogen) was included as previously published [35]. Standard conditions and experimental controls were performed as per manufacturer recommendations (Sialix). hiPSCs that were derived and maintained under xeno-free clinical grade conditions and mouse embryonic fibroblasts (Globalstem) served as negative and positive controls, respectively. Additionally, post-excised iPSCs in mTeSR1 plated on Matrigel and post-excised iPSCs in xeno-free NutriStem plated on CELLstart were utilized for this assay.

### Statistical analysis

Results are presented as means ± standard deviations. The statistical significance of differences for *PRPF39* gene expression was evaluated using SPSS 20 (IBM Corporation, Chicago, IL, USA). Analysis of variance, a *t* test for independent samples, and Kruskal–Wallis nonparametrical one-way analysis of variance tests were considered statistically significant with *P* <0.05.

## Results

### Induced pluripotent stem cell generation and characterization

**Figure 1:**
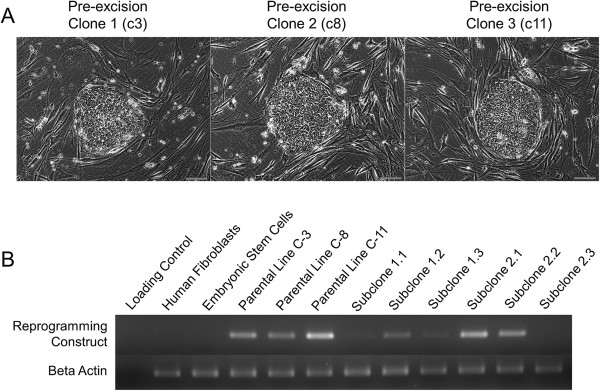
**Representative colonies from the three hSTEMCCA-derived induced pluripotent stem cell lines. (A)** Only the C8 pre-excised induced pluripotent stem cell (iPSC) line was found to have one integration into the *PRPF39* gene and was therefore selected to undergo Adeno-Cre-PuroR selection for removal of the cassette. **(B)** Excision of hSTEMCCA from the pre-excised iPSC adult parental line (C8). RT-PCR of genomic DNA with primers against hSTEMCCA elements endo-Myc-s and A-WPRE, showing that one subclone (2.3 post-excised iPSCs) was free of the integrated provirus. Bars = 100 μM.

Previous work has shown that adult somatic human dermal fibroblasts can be efficiently reprogrammed into iPSCs through exogenous expression of four transcription factors (OCT4, KLF4, c-MYC, and SOX2) with a single polycistronic lentivirus, or STEMCCA, flanked by loxP sites (hSTEMCCA-loxP) [16]. We reprogrammed low-passage adult human dermal fibroblasts through transduction of hSTEMCCA. To induce reprogramming, 1 × 10^5^ fibroblasts were transduced with a constitutively active hSTEMCCA-loxP. From these 100,000 cells, 60 colonies with ESC-like morphology were observed, providing a reprogramming efficiency of just over 0.05%, an efficiency which parallels that seen in the literature (Table 1). Twenty colonies were picked and iPSC lines were derived. The 16 iPSC lines with the best morphology were expanded and cryopreserved. Three iPSC lines were thawed and expanded for further analysis for this study. All three iPSC lines possessed typical human ESC-like morphology, including large nucleoli, a high nucleus-to-cytoplasm ratio, and tight compact colonies (Figure 1A). All three iPSC lines, which we define here as parental pre-excised C-3, C-8, and C-11, were originally cultured on MEF layers and standard ESC media conditions for over 20 passages, representing the most commonly used research-grade conditions for iPSC derivation and culture.

### Nonrestrictive linear amplification PCR genomic mapping of integration into *PRPF39* and pre-excised induced pluripotent stem cell characterization

Third-generation lentiviruses are capable of integrating into the host genome of primitive human repopulating cells multiple times, initially seeming to limit the practicality of using these viruses for reprogramming (for personalized cellular therapeutics) and warranting the need for new reprogramming methodologies that yield transductions with fewer copies per cell [44]. Optimization of the multiplicity of infection to between 0.1 and 10, however, recently demonstrated that over 94% of iPSC colonies had a single stable integration [16]. Extensive and site-specific genomic mapping to identify potential insertional mutagenesis and elucidate adverse gene expression effects is needed to establish therapeutically relevant and factor-free iPSC lines. To verify a single integrated STEMCCA line and specifically sequence and map the vector integration, nrLAM-PCR was used to analyze the vector-human genome location. Two lines (C3 and C8) demonstrated single intron-based integrations, and the third line (C11) demonstrated multiple integrations. The C3 iPSC line displayed one integration located in intron 5 of the lysosomal enzyme alpha-*N*-acetylgalactosaminidase (*NAGA*), and the C8 (pre-excised) iPSC line displayed one integration located in intron 4 of pre-mRNA-processing factor 39 (*PRPF39*), a protein known to interact with the spliceosome and play a role in pre-RNA processing [45]. Mutations in *NAGA* have been associated with Schindler disease [46], whereas mutations in *PRPF39* have not been correlated with any specific disease. We therefore focused our characterization and transcriptional analysis on the C8 line.

**Figure 2:**
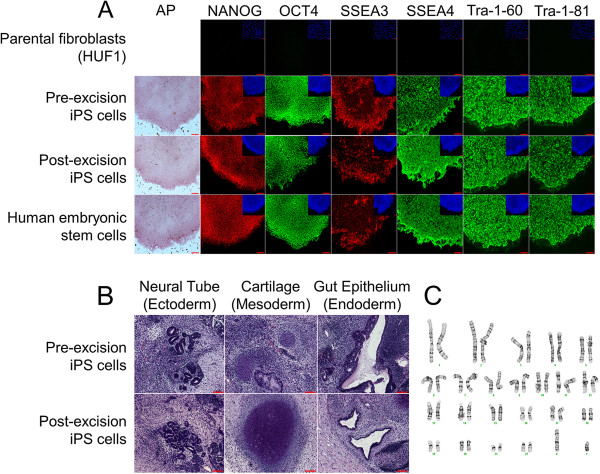
**Characterization of pre-excised and post-excised induced pluripotent stem cell lines. (A)** Expression of pluripotency markers from induced pluripotent stem cells (iPSCs) (human embryonic stem cells and parental fibroblasts from which the pre-excised iPSC line was derived serving as controls), showing similar expression of all markers before and after hSTEMCCA excision. **(B)** Histological analysis of teratomas derived from the pre-excised iPSC parental line and post-excised iPSC line. **(C)** G-band karyotyping analysis of the post-excised iPSC line, showing a normal 46XY karyotype following excision of hSTEMCCA. Bars = 100 μM.

The C8 pre-excised iPSC line expressed the pluripotency markers alkaline phosphatase, NANOG, OCT4, SSEA-3, SSEA-4, TRA-1-60, and TRA-1-81 as determined by immunocytochemistry (Figure 2A). Also, to demonstrate pluripotency, iPSCs were injected into the testes of a severe combined immunodeficient mouse. The pre-excised iPSC line successfully formed teratomas representative of all three germ layers: neural tube (ectoderm), gut epithelium (endoderm), and cartilage (mesoderm) (Figure 2B). These results demonstrate that our single integrated hiPSC line is pluripotent and able to contribute to representatives of all three germ layers.

### Adeno-Cre-puro excision

We next sought to generate a factor-free line void of any exogenous transgenic factors by expression of a nonintegrating adenovirus expressing both Cre-recombinase and puromycin resistance for selection of post-excised iPSC colonies. C3 and C8 iPSC cells were transduced for 24 hours with the Adeno-Cre-PuroR adenovirus and exposed to puromycin for 5 days, and then colonies were picked to establish three subclones from each colony (C3 subclones 1.1, 1.2, and 1.3 and C8 subclones 2.1, 2.2, and 2.3) after 2 weeks of recovery growth. We determined that successful Adeno-Cre-mediated excision of hSTEMCCA-loxP reprogramming construct occurred in only subclone 2.3, now called the post-excised iPSC subcloned line, as determined through PCR of genomic DNA with primers against endo-Myc-s and A-WPRE (Figure 1B). Expanded post-excised iPSCs were re-exposed to puromycin for 5 days, resulting in 100% cell death of all subcloned colonies and demonstrating that the Adeno-Cre-PuroR did not integrate into the genome following excision. Following Cre-mediated excision, post-excised iPSCs displayed a stable, uniform human ESC-like morphology for over 10 passages on MEFs and maintained pluripotent markers (alkaline phosphatase, NANOG, OCT4, SSEA-3, SSEA-4, TRA-1-60, and TRA-1-81) at a level comparable with that of the pre-excised iPSC line and control human ESCs (Figure 2A). Importantly, to avoid any MEF mRNA contamination issues in later applications, both the pre-excised and post-excised iPSC lines were transitioned into feeder-free conditions on Matrigel with mTeSR1 media. Post-excised cells also were able to maintain their pluripotency, as shown through their successful contribution to all three germ layers in teratoma formation (Figure 2B). Also, the post-excised iPSC line was able to maintain genomic stability for over 37 passages during the transition from pre-excised to post-excised hiPSCs as demonstrated by the normal karyotype maintained (Figure 2C). The completely factor-free post-excised iPSCs were therefore able to maintain pluripotency markers and a normal karyotype and to retain the ability to differentiate to representatives of all three germ layers in the teratoma assay.

### *PRPF39* gene expression and splicing analysis

**Figure 3:**
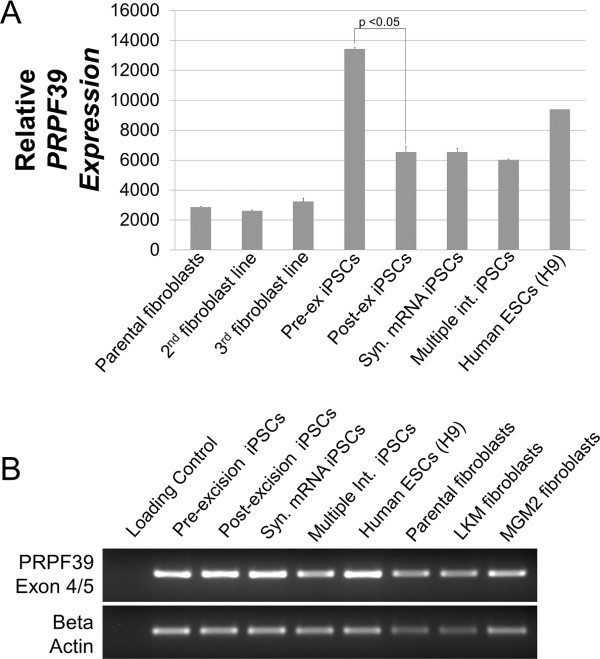
**Expression and splicing analysis of the pre-excised and post-excised lines. (A)** Quantitative PCR analysis showed that integration into the *PRPF39* gene caused a statistically significant increase in transcript expression, in which the increased expression was abrogated upon Adeno-Cre-PuroR-mediated hSTEMCCA excision. No statistical differences were seen between all control induced pluripotent stem cell (iPSC) lines, and all fibroblast lines displayed very low expression of *PRPF39*, indicating that this gene is associated with pluripotency. **(B)** To confirm that proper splicing of the transcript is taking place, primers against exons 4 + 5 show that no cryptic splice sites were being introduced and that, upon excision, the transcript correctly spliced itself. *P* <0.05. ESC, embryonic stem cell; Multiple Int., multiple integration; Syn., synthetic.

Successful excision of the hSTEMCCA-loxP site [16,47] and specific loci mapping of the virus integration were reported [31], but this study did not examine gene expression and splicing analysis of a post-excised hiPSC line in detail with sensitive real-time RT-PCR. We therefore sought to investigate the differential gene expression due to the lentiviral integration of hSTEMCCA-loxP into the integrated gene (that is, *PRPF39*). Even after excision, approximately 200 base pairs of exogenous DNA from the inactive long terminal repeat (LTR) remain integrated into the genome in intron 4 of *PRPF39*. This finding emphasizes the importance of gene expression analysis [48]. First, we performed microarray analysis on the pre-excised and post-excised iPSCs, and like previous investigators [31] we found no statistically significant difference between the gene expression for the integrated gene (*PRPF39*) (data not shown). Next, we used quantitative PCR to analyze the expression of *PRPF39* with exon-spanning primers across exons 2 and 3. The pre-excised iPSC line demonstrated a statistically significant increase in the expression of *PRPF39* compared with the post-excised iPSC line and every other cell line tested (Figure 3A). We confirmed this higher expression by running three different fibroblast lines as controls and showing very low expression of *PRPF39* in fibroblasts (Figure 3A). To investigate whether this expression difference was due to random insertional positional events in the genome, the multi-integration line iPSCs that had three integrations were also analyzed, and they yielded a nonstatistically significant difference with the post-excised iPSC line of *PRPF39* gene expression. This suggests that the expression difference of the pre-excised iPSC line is specifically due to the insertion of the hSTEMCCA-loxP viral construct into the *PRPF39* intron 4 and that, following excision, endogenous and homeostatic levels of *PRPF39* gene resumes. To further confirm correct splicing patterns, primers spanning exon 4 and 5 were used, and proper splicing was confirmed throughout all of the lines tested, including both pre-excised and post-excised iPSC lines (Figure 3B). Also, to confirm the splicing product homogeneity and proper PCR amplification, all lines tested were sequenced and yielded identical sequences. Importantly, although aberrantly increased *PRPF39* expression is seen in the pre-excised iPSC line, the post-excised line was able to revert back to normal levels of expression, indicating that the leftover LTR region was being properly removed during splicing.

### Differentiation into clinically relevant cell types

**Figure 4:**
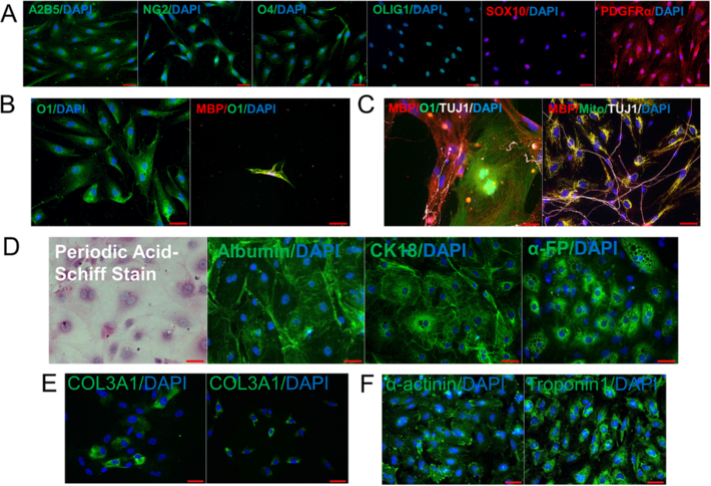
**Differentiation into therapeutically relevant cell lineages. (A)** Post-excised induced pluripotent stem cells (iPSCs) were differentiated into oligodendrocyte progenitor cells expressing characteristic progenitor stage markers. **(B)** Upon terminal differentiation, the progenitor cells matured and displayed the mature antigen O1 and also stained positive for secreting myelin basic protein (MBP), a hallmark of mature oligodendrocytes. **(C)** Due to low efficiency of *in vitro* oligodendrocyte maturation, a co-culture system with dorsal root ganglion neurons was utilized and showed mature oligodendrocytes intimately associated with, and myelinating, neurite outgrowths. Additionally, human mitochondria were stained to display that rat oligodendrocytes were not contaminating the culture. **(D)** Hepatocytes were derived that stained positively for glycogen synthesis as indicated by the periodic acid–Schiff stain, and CK18, albumin, and alpha-fetoprotein. **(E)** Derived fibroblasts stained positive for COL3A1 upon differentiation and at levels comparable with those of control fibroblasts (left picture is iPSC-derived fibroblasts and right are control fibroblasts). **(F)** Cardiomyocytes showed expression of alpha-actinin and Troponin 1. DAPI, 4′,6-diamidino-2-phenylindole. Bars = 50 μM.

To determine whether our transgene-free iPSC line was capable of differentiating into therapeutically relevant cell types, four different cell lineages were derived. First, oligodendrocyte progenitor cells (which could prove useful for treatment of spinal cord injuries) were differentiated and shown to express characteristic oligodendrocyte progenitor cell markers using immunocytochemistry. Oligodendrocyte progenitors express A2B5, NG2, OLIG1, SOX10, PDGFRα, and O4 (Figure 4A). After *in vitro* maturation, O1 and myelin basic protein were detected, indicating that we derived functionally mature oligodendrocytes (Figure 4B) [38]. However, it is well known that *in vitro* maturation is inefficient and minimal myelin production is produced without addition of further cytokines [38,49]; thus oligodendrocyte progenitor cell maturation is normally presented *in vivo*, where three-dimensional myelination formation is easier to achieve [50]. Therefore, to increase the efficiency of the *in vitro* model, the oligodendrocyte progenitor cells were co-cultured with rat DRG neurons to show an enhanced ability of the oligodendrocytes to produce myelin and myelinate DRG axons; human mitochondria were also stained to prove no contamination of rat oligodendrocytes in the co-culture (Figure 4C). This is an important step in developing an *in vitro* co-culture system that can allow oligodendrocytes to wrap around axons and display myelination capabilities. Second, functional hepatocytes were derived, as indicated by positive staining for glycogen synthesis following the periodic acid–Schiff test (which could prove useful for treatment of liver diseases such as urea cycle disorders). Cytokeratin 18, serum albumin, and alpha-fetoprotein also were localized with these hepatocytes (Figure 4D). Third, post-excised iPSCs were differentiated into fibroblasts (which could prove useful to generate large numbers of therapeutically useful autologous fibroblasts following gene correction, such as for Epidermolysis Bullosa) that stained positive for a characteristic fibroblast marker, Col3A1 (Figure 4E, left image), at levels comparable with those of control fibroblasts (Figure 4E, right image). Lastly, we derived cardiac myocytes [41] (which could be useful for treatment of heart disease) that were able to beat in culture [40] (see Additional file 1) and also stain positively for alpha-actinin and troponin1 (Figure 4F). Post-excised iPSCs therefore not only maintained pluripotency following hSTEMCCA-loxP excision and proper *PRPF39* expression levels, but also were able to differentiate into four functionally useful cell types that have direct therapeutic applications.

### Transition from research-grade to putative clinical-grade induced pluripotent stem cells

**Figure 5:**
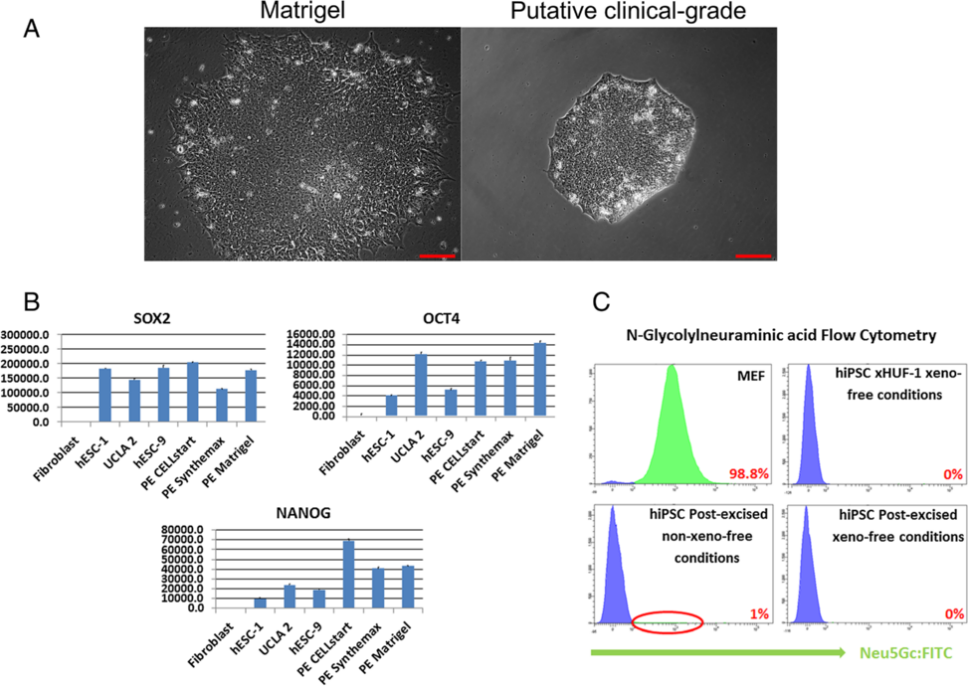
**Morphology of transgene-free induced pluripotent stem cells following conversion to clinical-grade conditions. (A)** Conversion of post-excised iPSCs from a xeno-containing substrate, Matrigel, to a xeno-free containing substrate, Synthemax, under current good manufacturing practice (GMP) conditions. **(B)** Quantitative PCR for pluripotency associated genes displays that pre-converted and post-converted induced pluripotent stem cells (iPSCs) retain normal expression levels across multiple synthetic substrates. **(C)** Beyond the standard GMP-grade sterility testing, a flow cytometry-based assay for a nonhuman antigen, *N*-glycolylneuraminic acid, displayed that upon GMP-grade conversion all sialic acid detection was eliminated (1% with post-excised cells on Matrigel down to 0% with post-excised cells in GMP conditions). An iPSC line derived under GMP conditions and mouse embryonic fibroblasts (MEFs) were used as negative and positive controls, respectively. hESC, human embryonic stem cell; hIPSC, human induced pluripotent stem cell; PE CELLstart, post-excised iPSC CELLstart; PE Synthemax, post-excised iPSC Synthemax; PE Matrigel, post-excised Matrigel.

We slowly transitioned our research-grade lines from a xeno-containing substrate and media to xeno-free conditions that maintained the pluripotent capability and functionality of the hiPSCs. Transitioning of post-excised iPSCs to xeno-free media conditions consisting of a 1:1 blend of NutriStem/TeSR2 was carried out over a period of 30 days, and this was considered a slow conversion methodology. After the post-excised iPSC line was stably passaging in the xeno-free media, the cells were passaged onto a xeno-free substrate called Synthemax and passaged multiple times, and only the best colonies were selected for each passage (Figure 5A). Next, we performed an extended cultivation (for more than 3 months) of our transgene-free iPSCs in defined xeno-free conditions (free from nonhuman serum, proteins, and cells) under current GMP manufacturing facilities (inspected and licensed by the state of California) and used qualified defined reagents and a standardized protocol [51]. The cells were also subjected to standardized quality-control testing to ensure viability, sterility, and appropriate cellular composition, including expression of standard stem cell markers (*NANOG*, *OCT4*, and *SOX2*) as indicated by quantitative PCR analysis (Figure 5B). We also converted the post-excised line from Matrigel and mTeSR1 to xeno-free and chemically defined CELLstart and NutriStem media under the same slow transition methodology as that used for the Synthemax and mTeSR1/NutriStem conversion. We used a different xeno-free substrate, and NutriStem alone, to show the robustness of the methodology across substrates and reagents. Both GMP grade conversions (Synthemax and CELLstart) yielded comparable expression of standard pluripotency markers (Figure 5B). Additionally, the pre-converted and post-converted iPSCs were tested, through flow cytometry, for the sialic acid/*N*-glycolylneuraminic acid (Neu5Gc), indicative of a nonhuman animal product contamination [52]. The iPSC line, xHUF-1, which was derived under completely defined and xeno-free conditions, and MEFs, which are of mouse origin, were used to show the negative and positive specificity of the antibody towards Neu5Gc, respectively. As expected, the MEF cells and the GMP iPSC line were 98.8% and 0% positive for Neu5GC, respectively (Figure 5C). We found that the post-excised line kept on Matrigel and in mTeSR1 media still possessed significant Neu5Gc, detectable on 1% of cells, but that this Neu5Gc was subsequently completely lost during the GMP conversion process. We did not define whether this 1% Neu5Gc detection was attributed to contaminating nonhuman epitopes from the original MEFs, serum and/or Matrigel. Regardless, this Neu5Gc detection approach is a stringent assay to determine iPSCs and their derivatives are free from animal epitopes that could lead to an immunogenic response [53].

## Discussion

In this study, we show successful derivation of hiPSCs from human adult somatic dermal fibroblasts that contain a single hSTEMCCA-loxP lentiviral integration. We used nrLAM-PCR technology to analyze both the number of integrations in each line and the site in the genome where the lentiviral provirus integrated. One pre-excised line was derived with a single integration found to map into intron 4 of *PRPF39* (a gene not associated with any disease). Following Adeno-Cre-PuroR-mediated excision, a factor-free line, termed post-excised iPSCs, was derived and propagated.

Because previous studies using the polycistronic human STEMCCA lentivirus did not analyze the expression and splicing patterns of an integrated and subsequently excised hSTEMCCA construct in detail, we sought to characterize the expression and splicing patterns of our post-excised iPSC line. Small inactive viral LTRs left in the genome are thought to cause a small risk of insertional mutagenesis [16]. A recent paper, however, argues that only transcriptionally active LTRs, and not transcriptionally inactive LTRs, are capable of forming myeloid tumors, even when multiple LTR copies are present [54]. Previous studies also showed that HIV-based vectors have a clear correlation between increased gene activity hotspots and integration site preference [28], although not specifically into transcriptional start sites as seen with retroviruses [54]. Therefore, despite the fact that oncogenic risk from an inactive LTR is low, the possibility of integration into a transcriptionally active location and gene is high, and therefore target gene expression and splicing data on the integration site are critical. Although we show abnormally increased gene expression in the pre-excised iPSC line, the gene expression levels were reduced to basal levels upon excision, and the post-excised line maintained a normal pluripotent stem cell phenotype. Fortunately, the post-excised iPSC line had proper splicing of *PRPF39* mRNA, although this is probably due to the wild-type nonintegrated allele properly expressing *PRPF39*. It is important to show that the lentiviral integration does not cause dominant negative interactions with the wild-type allele, allowing normal expression. *PRPF39* gene expression was therefore increased, probably due to an enhancer element like the woodchuck post-transcriptional regulatory element coded by the lentivirus causing a post-transcriptional increase in gene expression [55]. If current safe-harbor criteria are expanded to include intron-based reprogrammed cells that have been characterized to demonstrate a normal post-excision integrated gene expression profile, such as the cells described in this study, this will increase the proportion of generated iPSC lines being considered for therapeutic applications and thereby increase the feasibility of iPSC-based therapeutics. We also demonstrated that the post-excised iPSC line was able to differentiate into multiple therapeutically important cell types, such as hepatocytes, cardiomyocytes, and oligodendrocyte progenitor cells [56].

Finally, we sought a regulatory path to convert these research-grade transgene-free hiPSCs into cells that could be used in future clinical therapeutics (clinical grade). This transition from research grade to clinical grade was previously performed for human ESCs initially derived in the presence of nonhuman serum, proteins, and cells [57]. Geron converted their research-grade ESCs to clinical-grade ESCs by extended cultivation of their cells in defined xeno-free conditions free from nonhuman serum, proteins, and cells under current GMP manufacturing facilities. These facilities involve clean-room suites that are inspected and licensed by the state of California and use of qualified defined reagents and a standardized protocol, followed by standardized quality-control testing. In this study, we used the same approach and converted our research-grade transgene-free iPSCs into putative clinical-grade iPSCs. We discovered that while a small percentage (1%) of the research-grade cells still demonstrated detectible nonhuman sialic acid (which may induce an immunogenic response if these cells had been used for autologous cellular therapeutics [53]), the post-converted cells no longer demonstrated any detectible sialic acid, suggesting that these cells were now clean and could be used without risking an immunogenic response. However, several caveats must be kept in mind. First, the previous US Food and Drug Administration-approved conversion of research-grade human pluripotent stem cells to clinical-grade cells involved ESCs, not iPSCs, and it is not guaranteed that the same conversion criteria will apply for iPSCs. Second, the US Food and Drug Administration approval technically applied to one specific derivative (oligodendrocyte precursor cells) derived from the converted clinical-grade ESCs, and suggests that a separate US Food and Drug Administration approval would be required for each iPSC-derived, differentiated therapeutic product. How the US Food and Drug Administration will ultimately judge the clinical applicability of these iPSCs, their derivatives, and other future iPSC-based therapeutics initially derived under research-grade xeno-containing conditions remains to be determined.

## Conclusions

In summary, we have demonstrated the derivation of a factor-free hiPSC line using a polycistronic human STEMCCA reprogramming virus. nrLAM-PCR-based genomic mapping showed that the line had a single integration into a relatively safe location in intron 4 of the *PRPF39* gene. We then demonstrated proper expression levels following excision of the viral construct, correct splicing patterns, differentiation of the post-excised iPSCs into therapeutically relevant cell lineages, and transition into putative clinical-grade conditions.

## Abbreviations

DMEM: Dulbecco’s modified Eagle’s medium; DRG: Dorsal root ganglion; EB: Embryoid body; ESC: Embryonic stem cell; FBS: Fetal bovine serum; GMP: Good manufacturing practice; H & E: Hematoxylin and eosin; hiPSC: Human induced pluripotent stem cell; iPSC: Induced pluripotent stem cell; MEF: Mouse embryonic fibroblast; miRNA: MicroRNA; nrLAM: Nonrestrictive linear amplification; PBS: Phosphate-buffered saline; PCR: Polymerase chain reaction; PSC: Pluripotent stem cell; RT: Reverse transcription; STEMCCA: Stem cell cassette.

## Competing interests

JAB and AV-C receive research funding for Fibrocell Science, Inc. JAB is a scientific consultant for Fibrocell Science, Inc. The remaining authors declare that they have no competing interests.

## Authors’ contributions

JAB and JPA designed the experiments and wrote the manuscript, JPA, PCL, CR, AV-C, and SK performed the iPSC derivation and characterization experiments. JPA, PCL, CR, JD-D and AC performed the iPSC analysis experiments. MGM derived and analyzed the fibroblasts. PEP assisted with the oligodendrocyte characterization assays and GSL assisted with the hepatocyte characterization assays. ADP and ATC assisted with teratoma formation assays and analysis. VS and RAR directed the initial reprogramming efforts. SK, WEL, JAZ, DBK, ADP, ATC and JAB directed the establishment of the UCLA GMP facilities permitting the conversion and characterization of clinical-grade iPSCs. All authors contributed to the final draft of manuscript.

## Supplementary Material

Additional file 1 Is a video file showing spontaneous differentiation of transgene-free iPSCs into functional beating cardiomyocytes. Click here for file
